# MHz X-ray photon correlation spectroscopy using an acoustic levitator at the European XFEL

**DOI:** 10.1107/S1600577525002875

**Published:** 2025-04-25

**Authors:** Wonhyuk Jo, Johannes Möller, Jörg Hallmann, James Wrigley, Jan-Etienne Pudell, Ulrike Boesenberg, Felix Brausse, Angel Rodriguez-Fernandez, Alexey Zozulya, Roman Shayduk, Anders Madsen

**Affiliations:** a European X-ray Free-Electron Laser Facility, Holzkoppel 4, 22869Schenefeld, Germany; University College London, United Kingdom

**Keywords:** X-ray photon correlation spectroscopy, acoustic levitator, X-ray free-electron lasers, colloidal nanoparticles, coherence speckle

## Abstract

The structural and dynamical properties of soft-matter systems play an important role in crystallization and nucleation theory. Despite their significance, the dynamical properties are still poorly understood because of experimental constraints and the requirement of performing measurements with high spatial and temporal resolution. Here, we demonstrate MHz X-ray photon correlation spectroscopy (XPCS) using a contactless sample holder at the European X-ray Free-Electron Laser. A millimetre-sized liquid sample droplet was levitated in air via acoustic waves with the solvent slowly evaporating. A colloidal suspension of silica nanospheres was used to track the structural evolutions using small-angle X-ray scattering, and the dynamical information was captured by time-resolved MHz XPCS as a function of evaporation time. This study outlines a new path towards the investigation of metastable structure and dynamics using X-ray speckle techniques, for instance, XPCS, X-ray speckle visibility spectroscopy and X-ray cross-correlation analysis.

## Abstract 2

MHz X-ray photon correlation spectroscopy with a containerless sample holder at the European XFEL is demonstrated.

## 1. Introduction

Containerless or contactless sample environments have been widely employed to investigate the thermophysical properties of metastable states, which play an important role in many phase transformations, and it is essential to eliminate container walls that otherwise can lead to unwanted nucleation or pinning effects. The absence of containers suppresses the heterogeneous nucleation process and sustains metastable states, and might give a unique opportunity to probe such delicate systems experimentally. Different containerless methods, so-called levitation techniques, have been developed, for instance electrostatic levitators (ESLs) (Lee *et al.*, 2017 ▸; Jeon *et al.*, 2022 ▸), electromagnetic levitators (EMLs) (Matson *et al.*, 2023 ▸), aerodynamic levitators (Langstaff *et al.*, 2013 ▸) and acoustic levitators (ALs) (Aoki & Hasegawa, 2020 ▸), to study bulk metallic glasses (Mukherjee *et al.*, 2004 ▸), ionic solutions (Lee *et al.*, 2016 ▸; Hwang *et al.*, 2021 ▸; Cho *et al.*, 2024 ▸), polymorphic transitions (Gnutzmann *et al.*, 2014 ▸), chemical manipulation (Watanabe *et al.*, 2018 ▸) and colloidal particles (Hwang *et al.*, 2020 ▸). Consequently, the measurements also need to be done without physical contact, so light-based techniques, such as Raman and X-ray scattering, are the most relevant tools to conduct these experiments. Earlier work has primarily focused on the evolution of the atomic or molecular structure because of experimental difficulties in acquiring dynamical information, for instance governing the nucleation rate and crystal growth mechanism. The levitators generate an external force to offset the gravitational force and operate with a certain frequency (*e.g.* 150 Hz for ESL and 23 kHz for AL) to stabilize the sample position on a macroscopic scale. However, sample jittering is unavoidable on a microscopic scale, which will complicate the interpretation of dynamical measurements without sufficient temporal resolution (Derkachov *et al.*, 2020 ▸).

X-ray photon correlation spectroscopy (XPCS) (Grübel & Zontone, 2004 ▸; Sutton, 2008 ▸; Madsen *et al.*, 2010 ▸) is a very powerful and well established coherent X-ray scattering technique for investigating the atomic and molecular dynamics of complex disordered systems such as colloids (Thurn-Albrecht *et al.*, 1996 ▸; Fluerasu *et al.*, 2007 ▸; Li *et al.*, 2014 ▸; Jo *et al.*, 2021 ▸), polymers (Czakkel & Madsen, 2011 ▸; Lehmkühler *et al.*, 2018 ▸; Frenzel *et al.*, 2019 ▸; Reiser *et al.*, 2022*a* ▸), capillary waves (Seydel *et al.*, 2001 ▸), metallic glasses (Ruta *et al.*, 2012 ▸; Evenson *et al.*, 2015 ▸), molecular glasses (Chushkin *et al.*, 2012 ▸) and water (Perakis *et al.*, 2017 ▸). The temporal resolution of XPCS is defined by the number of speckle images that can be collected per unit time, so both the X-ray flux [and repetition rate for XPCS at pulse-based sources like free-electron lasers (FELs)] and detector frame rate are crucial parameters. The European X-ray Free-Electron Laser (XFEL), providing an X-ray repetition rate of up to 4.5 MHz (Tschentscher *et al.*, 2017 ▸; Decking *et al.*, 2020 ▸), and the Adaptive Gain Integrated Pixel Detector (AGIPD) (Allahgholi *et al.*, 2019 ▸; Sztuk-Dambietz *et al.*, 2024 ▸) have extended the temporal resolution down to a few hundred nanoseconds, hence enabling the investigation of complex polymer and protein dynamics on the microsecond scale (Lehmkühler *et al.*, 2020 ▸; Dallari *et al.*, 2021 ▸; Reiser *et al.*, 2022*b* ▸; Dallari *et al.*, 2024 ▸).

In this paper, we demonstrate the feasibility of MHz XPCS with an AL at the European XFEL to investigate the structure and dynamics of colloidal particles in water. The AL enables contactless levitation of a millimetre-sized liquid droplet in air, where solvent evaporation naturally leads to a gradual increase in particle volume fraction. We have successfully developed a data evaluation method that accounts for the spatial jittering of the droplet, supported by simulations of dynamics. Our results show diffusion coefficients consistent with theoretical expectations, confirming the applicability of AL-based XPCS experiments at XFELs. This approach not only expands the capabilities of XPCS for studying colloidal dynamics but also provides a versatile platform for investigating metastable states and non-equilibrium processes in soft matter systems.

## 2. Experiment

**Figure 1:**
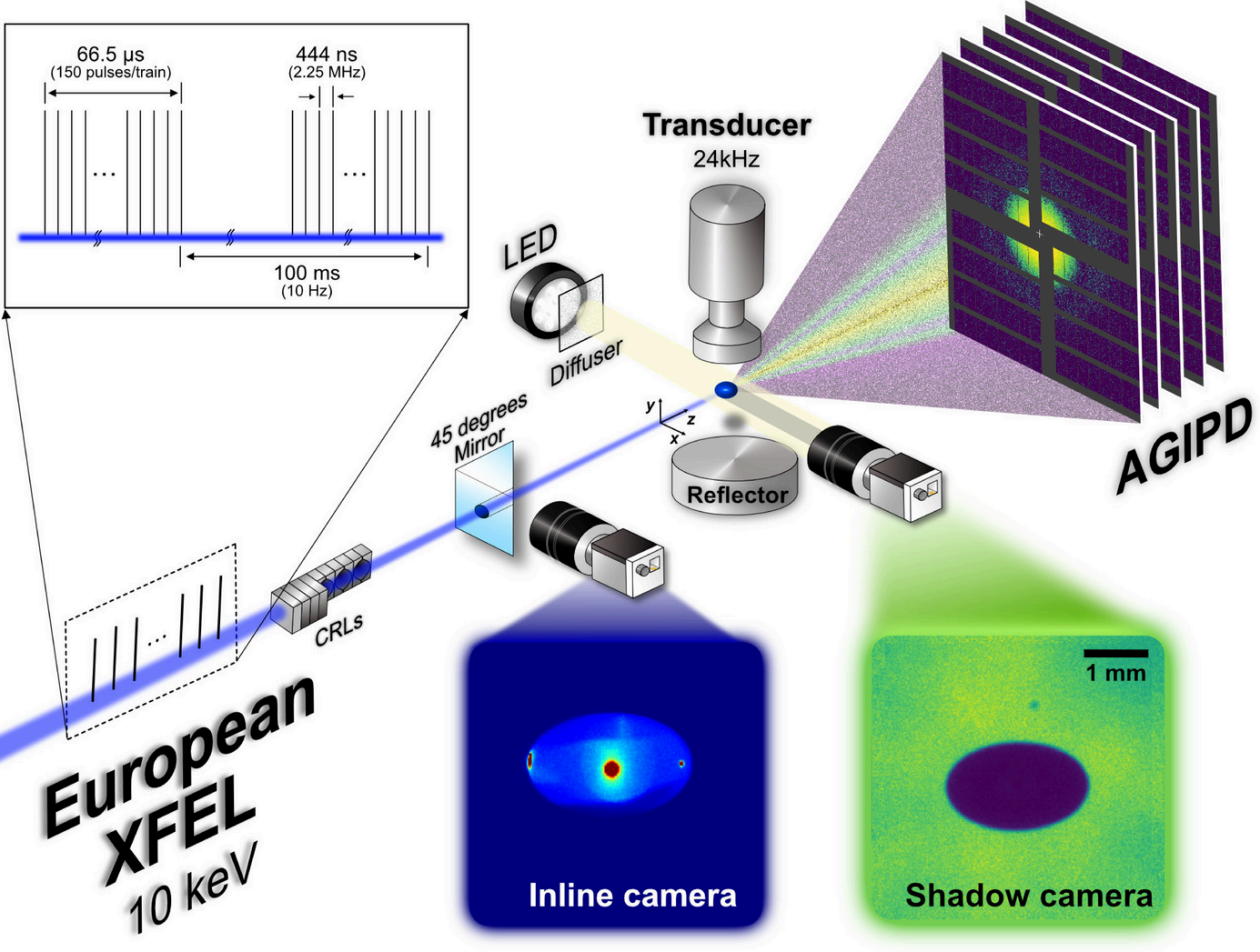
Experimental sketch of the acoustic levitator setup at the MID instrument. A total of 150 X-ray pulses temporally separated by 444 ns are hitting the levitated droplet in a train. The X-ray speckle patterns were recorded by the AGIPD and further processed to calculate the temporal auto-correlation functions. The inline camera shows light emitted from a droplet containing X-ray fluorescent powder.

The experiment was conducted at the Materials Imaging and Dynamics (MID) instrument (Madsen *et al.*, 2021 ▸) of the European XFEL using a photon energy of 10 keV (see Fig. 1 ▸). The self-amplified spontaneous emission (SASE) (Geloni *et al.*, 2010 ▸) X-ray beam was focused vertically and horizontally to a size *d*_b_ (diameter) of 10 µm using beryllium compound refractive lenses (CRLs) located 28 m upstream of the sample position. The repetition rate of the X-ray pulses in a train was 2.25 MHz, and the trains that were delivered at 10 Hz each contained 150 X-ray pulses. The AGIPD, of ∼1 Megapixel (1024  × 1024) and synchronized to the X-ray pulse pattern, was located at *L* = 7 m downstream of the sample to collect speckle patterns in the small-angle X-ray scattering (SAXS) regime, providing a maximum wavevector transfer of *q*_max_ = 0.112 Å^−1^. The speckle size was estimated according to *d*_s_ = λ*L*/*d*_b_ = 86.73 µm, where λ is the X-ray wavelength. The expected speckle contrast β taking into account the detector pixel size of 200 µm is about 3% in the *q* range of 0.01 Å^−1^ (Madsen *et al.*, 2016 ▸; Möller *et al.*, 2019 ▸). A liquid droplet was levitated at an acoustic pressure node generated by the AL. The distance between the acoustic transducer (UP400St, Hielscher Ultrasound Technology), generating a 24 kHz sound wave, and the reflector was optimized to form a standing wave. A transducer power of 120 W was applied to levitate a liquid droplet of ∼2 mm diameter. Water evaporates continuously from the droplet so a reduction in volume is seen with time. This increases the concentration of colloids accordingly. Every pulse generated a SAXS pattern at the detector, and approximately 2000 pulse trains (200 s) were recorded in a single run and repeated as long as the AL kept the sample levitated. Two observation cameras, *i.e.* inline and shadow image cameras, collected macroscopic droplet images at 10 Hz. The inline camera aligned with the 45° mirror observes the droplet image along the X-ray beam direction, see Fig. 1 ▸. The AL device was placed on a hexapod, allowing adjustment of the droplet position to be hit with the beam. Gd_2_O_2_S:Tb fluorescent powder dispersed in water was levitated as a droplet and used as a test sample for precise alignment. An LED backlight was employed for the shadow images to estimate the droplet volume as a function of evaporation time.

## 3. Result

### 3.1. Evaporation rate

**Figure 2:**
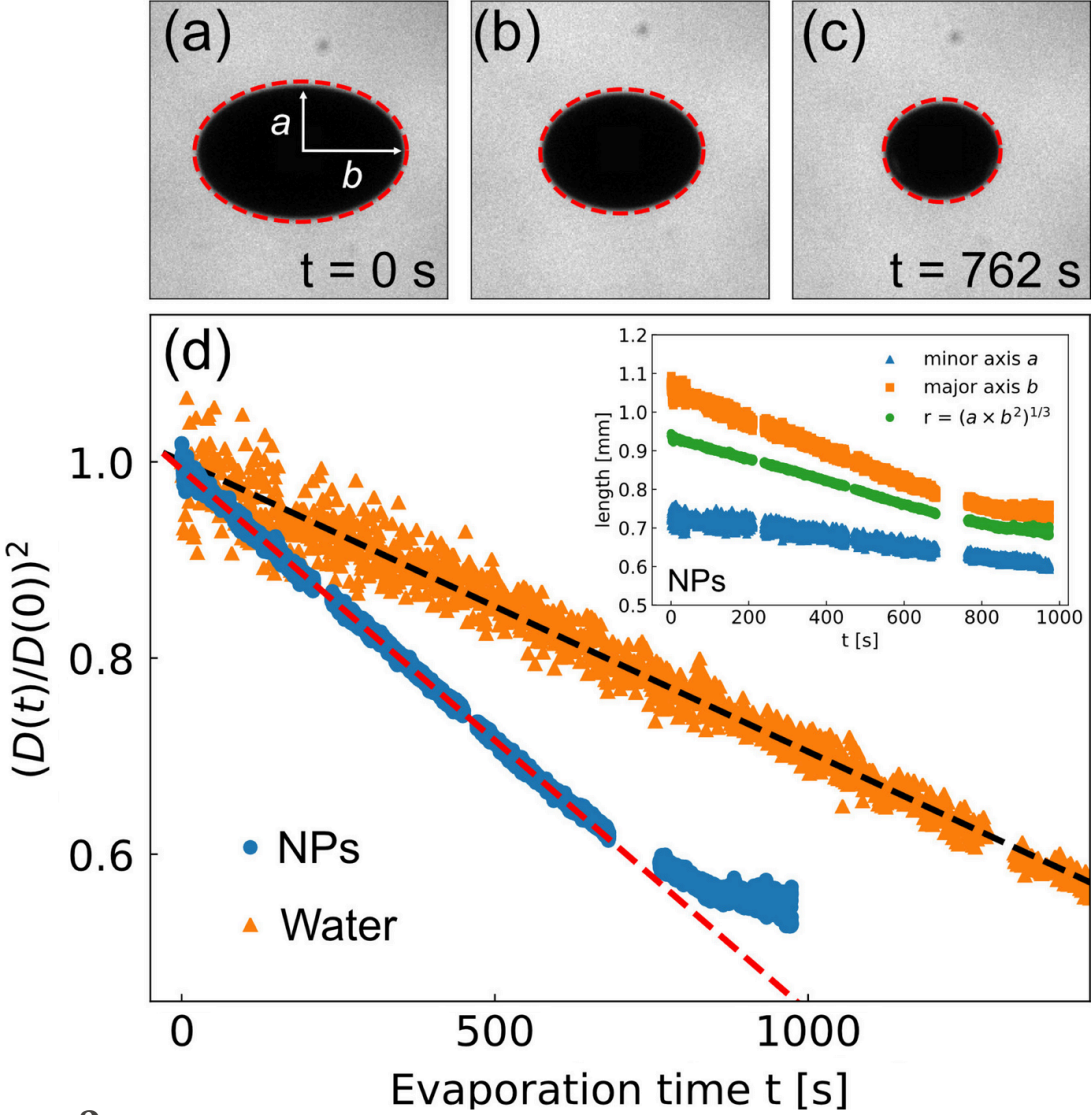
(*a*)–(*c*) The shadow images of droplets and the corresponding ellipsoidal fitting lines at different evaporation times. (*d*) Droplet diameter *D* = 2*r* as a function of evaporation time *t* estimated by the fitting result (inset). The dashed lines represent the corresponding fits of equation (1).

Charge-stabilized silica nanoparticles (Ludox TM-50, Sigma-Aldrich) were diluted with additional deionized water to reduce the volume fraction ϕ to 0.83%, which suppresses the particle interactions, hence leading to simple Brownian motion. The initial droplet volume was about 4 µl governed by the acoustic pressure distribution and the sample surface tension (Aoki & Hasegawa, 2020 ▸). Edge detection and ellipsoidal fitting scripts written in Python were employed to the shadow images in order to estimate the droplet volume [see Figs. 2 ▸(*a*)–2(*c*)]. The average diameter was estimated based on the length of the major and minor axes obtained from fitting of the images [inset of Fig. 2 ▸(*d*)]. The *D*^2^ law, which is based on a commonly accepted theory of model droplet evaporation, was employed to estimate the evaporation rate *K* as (McGaughey & Ward, 2002 ▸) 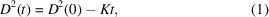
where *D* and *t* are the droplet diameter and evaporation time. Fig. 2 ▸(*d*) shows the *D*^2^ values obtained from the colloidal nanoparticle suspension (*i.e.* NPs) and pure water as a function of *t* with the fitting result according to equation (1). The obtained *K*_NPs_ and *K*_water_ are 2.03 × 10^−1^ and 1.38 × 10^−1^ mm^2^ s^−1^, respectively. We attribute the considerably higher evaporation rate of NPs compared to pure water with the X-ray pulses irradiating the NPs sample during the measurements. The water droplet evaporation was measured without illuminating X-rays. The high intensity of the European XFEL pulses induces thermal energy to the colloidal samples on microsecond time scales (Lehmkühler *et al.*, 2020 ▸), which enhances the evaporation process. We attribute the deviation from the *D*^2^ law that happens in the NPs sample at around 750 s [see Fig. 2 ▸(*d*)] to the increasing volume fraction of nanoparticles that changes the surface characteristics and hinders evaporation.

### 3.2. SAXS

**Figure 3:**
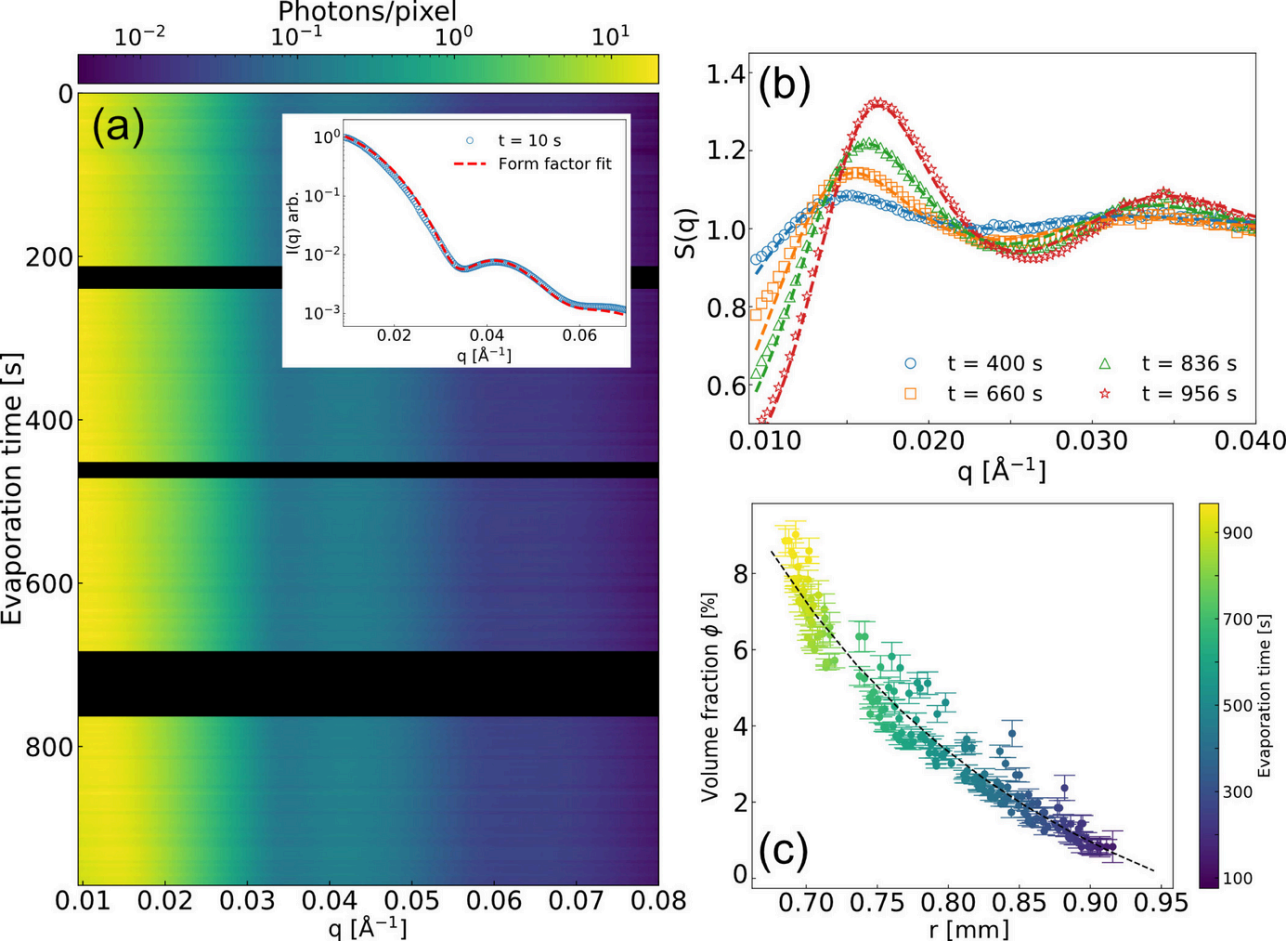
(*a*) Azimuthal integrated intensity profiles *I*(*q*) of silica nanoparticles dispersed in water. Each *I*(*q*) is the averaged result of 150 X-ray pulses. The inset figure shows the initial intensity profile together with a spherical form-factor fit yielding *R* = 12.94 nm. (*b*) Structure factor *S*(*q*) for varying evaporation time and the corresponding fits of the RMSA. (*c*) The volume fractions ϕ as a function of droplet radius *r*. The dashed line is a model fit with a ϕ ∝ 1/*r*^3^ law.

The structural evolution of colloidal nanoparticles was derived from the SAXS patterns recorded by the AGIPD (see Fig. 1 ▸). Fig. 3 ▸(*a*) shows the azimuthally integrated intensity distributions *I*(*q*) after averaging over the 150 X-ray pulses delivered in a train. The thick black horizontal lines around 210, 420, and 720 s represent time gaps between the measurements. The inset of Fig. 3 ▸(*a*) shows the normalized *I*(*q*) at *t* = 10 s together with a spherical form-factor *F*(*q*) fitting (Pedersen, 1997 ▸). The averaged particle radius *R* is 12.94 ± 0.04 nm, and the polydispersity Δ*R*/*R* is 8.79%. The structure factor *S*(*q*) was obtained by *S*(*q*, *t*) = *I*(*q*, *t*)/*F*(*q*) and is presented in Fig. 3 ▸(*b*) for varying evaporation times. A rescaled mean spherical approximation (RMSA) (Hansen & Hayter, 1982 ▸) and the hard-sphere Yukawa (HSY) fluid model (Heinen *et al.*, 2011 ▸) were employed to fit *S*(*q*) and estimate the volume fraction ϕ of the NPs sample as a function of evaporation time. The volume fractions of nanoparticles in the sample appear to increase from 0.83% to 9% and the droplet radius changed from 0.94 mm to 0.69 mm during the 960 s of total evaporation times. The dashed line in Fig. 3 ▸(*c*) represents fitting with a ϕ ∝ 1/*r*^3^ law.

### 3.3. XPCS

**Figure 4:**
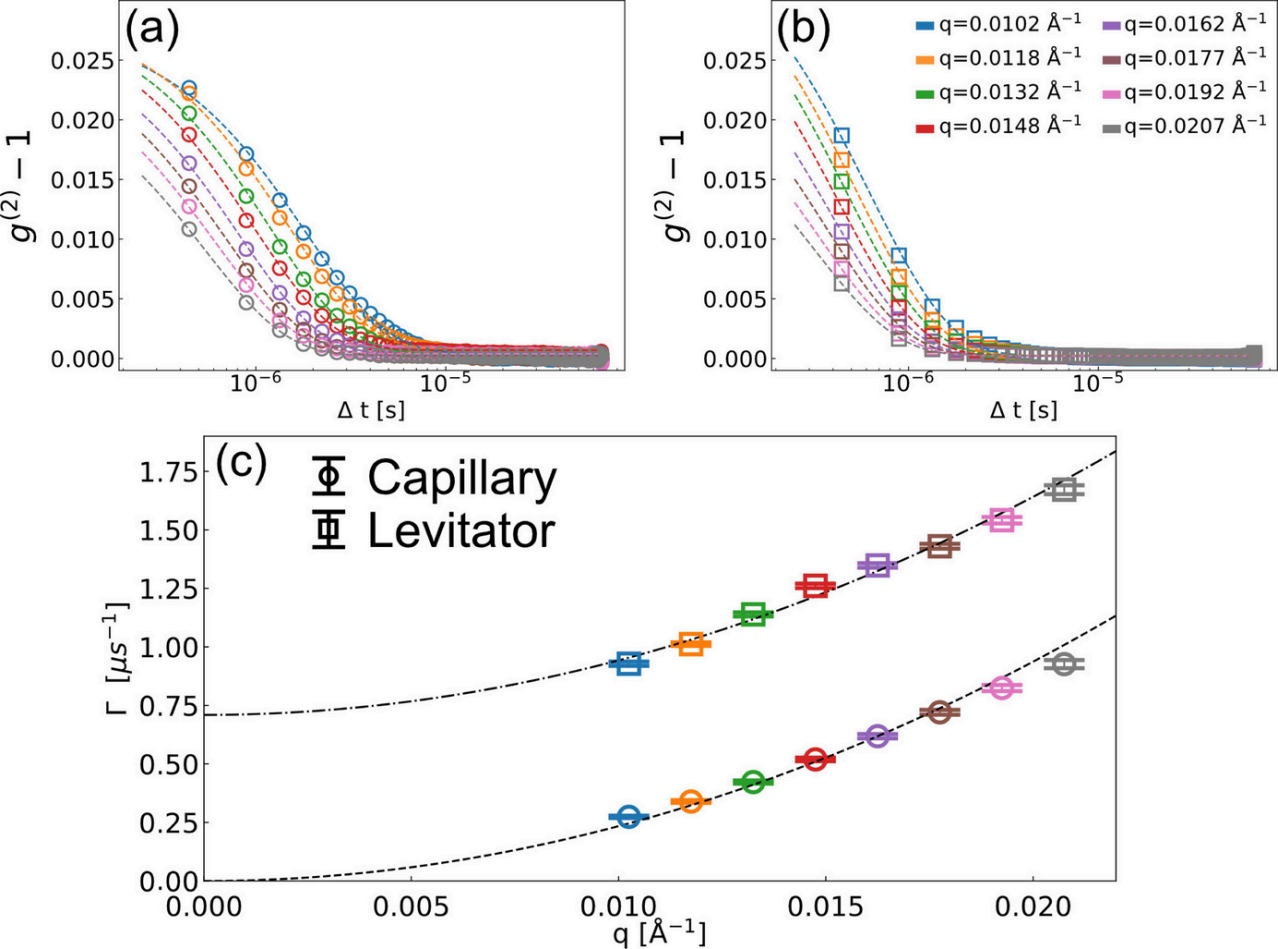
Intensity correlation functions obtained from silica nanoparticles measured in (*a*) a capillary and (*b*) a levitated droplet for varying momentum transfers *q*. Panels (*a*) and (*b*) share the color labels presented in (*b*) and the dashed lines are the fit result of equation (2). (*c*) The relaxation rate Γ as a function of *q*. The dashed and dash-dotted lines are the fit results of Γ = *D*_0,c_*q*^2^ and Γ = *D*_0,L_*q*^2^ + α, respectively.

The dynamics of colloidal nanoparticles were investigated by XPCS. The intensity fluctuations of the speckle patterns appear as a consequence of particle diffusion, hence enabling the dynamics to be described via the intensity auto-correlation function *g*^(2)^ and the Siegert relation (Siegert, 1943 ▸; Ferreira *et al.*, 2020 ▸) defined as 
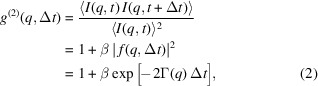
where *I*(*q*, *t*) represents the intensity of the speckle pattern at wavevector *q* and time *t*. The angled brackets 〈…〉 indicate time averaging. The wavevector *q* is defined by λ and the scattering angle 2θ according to *q* = 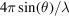. The speckle contrast β is determined by the experimental setup and configuration, such as the coherence properties, the sample thickness and the scattering geometry (Möller *et al.*, 2019 ▸; Lehmkühler *et al.*, 2021 ▸). The minimum delay time Δ*t* defines the temporal resolution, which in this case is 444 ns, corresponding to the X-ray repetition rate. The intermediate scattering function (ISF) is assumed to be a simple exponentially decaying function like *f*(*q*, Δ*t*) = exp[−Γ(*q*)Δ*t*] in a hard sphere monodisperse colloidal system. The relaxation rate Γ(*q*) is particularly simple for Brownian motion (free diffusion): Γ(*q*) = *D*_0_*q*^2^, where *D*_0_ is the Stokes–Einstein diffusion coefficient of the particles in the solvent. Fig. 4 ▸ shows the correlation functions for varying wavevector *q* obtained from the same silica NPs sample using two different experimental setups: (*a*) conventional capillary measurement and (*b*) using the acoustic levitator. Fig. 4 ▸(*c*) shows the corresponding relaxation times as a function of *q*. In the conventional measurement, the quartz capillary containing the sample was continuously moved during the measurement to probe a fresh volume for every pulse train. The relaxation rates Γ were obtained from the fitting using equation (2) and are presented in Fig. 4 ▸(*c*) as circles. The diffusion coefficient from the capillary measurements *D*_0,c_ = 23.42 ± 0.45 µm s^−1^ was estimated by least-squares fitting of Γ(*q*) = *D*_0_*q*^2^. In the simple silica nanoparticle system, the hydrodynamic radius *R*_H_ is assumed to be nearly equivalent to the particle radius *R* evaluated from the SAXS measurement shown in the inset of Fig. 3 ▸(*a*). The temperature *T* was numerically evaluated to be 306 K by the Stokes–Einstein relation as η(*T*)/*T* =*k*_B_/(6π*D*_0_*R*_H_), where *k*_B_, *T* and η are the Boltzmann constant, environment temperature and the solvent viscosity, respectively. For the numerical solution, the viscosity η was interpolated from tabulated values (Korson *et al.*, 1969 ▸), yielding 0.74 m Pa s. The slightly higher sample temperature than room temperature can be explained by the X-ray-induced heating effect within the train (Lehmkühler *et al.*, 2020 ▸). For the data evaluation of the levitation measurement in Fig. 4 ▸(*b*), the sample spatial jittering was considered. We investigated the results of single-train XPCS measurements and analyzed the diffusion coefficients. A Gaussian fit was applied to the histogram of diffusion coefficients, and we used the full width at half-maximum (FWHM) as a filter criterion. This selection process resulted in approximately 70% of trains being retained for analysis. The filtered-out trains are assumed to correspond to measurements taken under suboptimal conditions, such as instances where sample spatial jittering occurs faster than the sample characteristic time τ_c_ = Γ^−1^ or where complex jittering motions are notably present. For the silica NPs dispersed in water, we have focused on the earlier evaporation time (*t* < 200 s) when the particle concentration is still low and the sample hence should undergo free diffusion. After filtering, the calculated *g*^(2)^ functions show simple exponential decaying behaviors, and the fitted relaxation rate Γ also shows a clear *q*^2^ dependence in Fig. 4 ▸(*c*). However, the obtained dynamics are clearly faster than obtained in the capillary measurement on the same sample. In order to fit the relaxation rates, a constant offset α is required so Γ = *D*_0_*q*^2^ + α. With the constant α applied, the diffusion coefficient using the levitator *D*_0,L_ was estimated to be 23.29 ± 1.24 µm^2^ s^−1^ which is in excellent agreement with *D*_0,c_ together with α of 0.71 ± 0.028 µs^−1^.

## 4. Discussion

We attribute the constant offset α in Fig 4 ▸(*c*) to the levitated droplet’s spatial jittering during the X-ray train. The applied frequency of the acoustic transducer of 23 kHz may perturb the sample at least every 43 µs according to its period. In addition, airflow around the sample also affects the stability. In previous studies, when the sample characteristic time τ_c_ = 1/Γ is fast enough compared with the sample transit time *t*_transit_ = *a*/*v*, where *a* and *v* are the beam size and movement speed, respectively, this effect was negligible (Fluerasu *et al.*, 2008 ▸). Therefore, translating the sample with sufficient but not too high speed has been generally accepted in XPCS measurements as a method to ensure that every new pulse train was hitting a new sample volume, for instance to avoid radiation damage at the European XFEL. However, when *t*_transit_ is comparable with τ_c_, the movement interferes with the measured correlation functions. We have conducted two-dimensional simulations (*x* and *y*) to investigate the correlations between *t*_transit_ and Γ. Note that motion along the *z* direction (along the beam) is not supposed to affect the relaxation times even though the beam size on the sample can vary (Lurio *et al.*, 2021 ▸). Brownian motion of non-interacting particles was simulated and two assumptions were made: (1) the macroscopic droplet movement is collective, so the nanoscale particle dynamics is independent of the droplet motions; (2) the droplet movement had a constant speed with *v*_*x*_ and *v*_*y*_ during the simulation time. The simulations of nanoparticle motion were performed in three different scenarios:

(*a*) Brownian motion: the colloidal particles randomly move in the *x* and *y* directions mimicking free diffusion.

(*b*) Drift motion: the colloidal particles are in a static state, but the entire system moves with *v*_*x*_ and *v*_*y*_ to follow the spatial jitter of the droplet.

(*c*) Combined motion: the particles experience Brownian motion on top of the drift motion.

**Figure 5:**
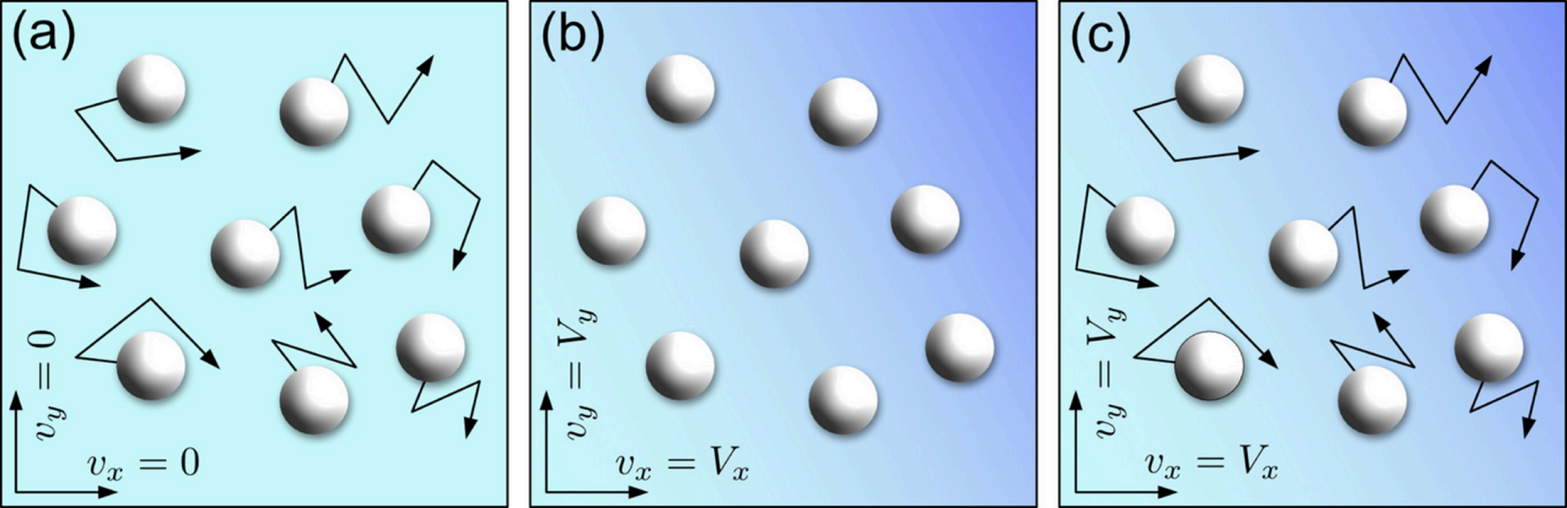
Conceptual sketches of the three simulation scenarios, which are (*a*) Brownian, (*b*) drift and (*c*) combined motion. The color gradients in the background of (*b*) and (*c*) represent the conceptual drift motions in the *x* and *y* directions.

Initially, one would expect that the dynamics from capillary and levitation measurements could be explained by Brownian and combined motion, respectively. The conceptual sketches are shown in Figs. 5 ▸(*a*), 5(*b*) and 5(*c*) for Brownian, drift and combined motion, respectively.

**Figure 6:**
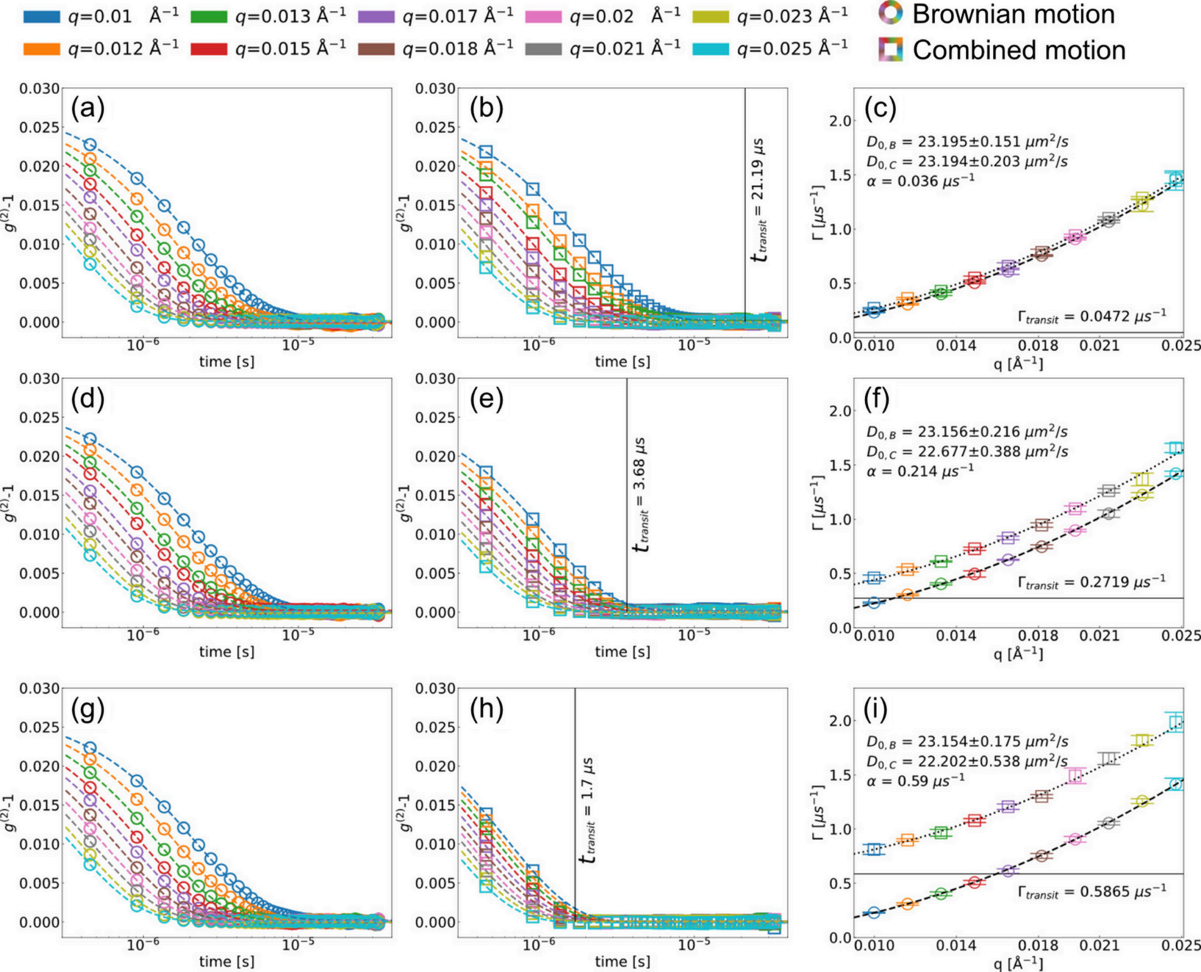
Simulation results for three translation speeds, *i.e.* slow (*a*)–(*c*), intermediate (*d*)–(*f*), and fast (*g*)–(*i*). *g*^(2)^ of the Brownian motions (*v* = 0) are shown in (*a*), (*d*) and (*g*), and *g*^(2)^ of the combined motions are shown in (*b*), (*e*) and (*h*). The dashed lines are the fit results of equation (2). The fitted values of Γ are shown in (*c*), (*f*) and (*i*). The dashed and dotted lines show the fitting curves of Γ = *D*_0_*q*^2^ and Γ = *D*_0_*q*^2^ + α, respectively.

In our simulation, three different drift speeds, *i.e.* slow (0.472 m s^−1^), intermediate (2.72 m s^−1^) and fast (5.87 m s^−1^), were employed, and the corresponding results are shown in Figs. 6 ▸(*a*)–6(*c*), 6(*d*)–6(*f*) and 6(*g*)–6(*i*), respectively. We employed only *y* directional drift motion (*v*_*x*_ = 0) for simplification. The resulting *g*^(2)^ and fitted Γ are shown as circles and squares for Brownian and combined motion, respectively. Note that we used Γ = *D*_0_*q*^2^ and Γ = *D*_0_*q*^2^ + α to extract the diffusion coefficient for Brownian (*D*_0,B_) and combined (*D*_0,C_) motions. In the simulation of Brownian motion, the obtained diffusion coefficients *D*_0,B_ are almost similar since the simulation conditions were identical.

In contrast, we found that the transit time *t*_transit_ [black vertical lines in Figs. 6 ▸(*b*), 6(*e*) and 6(*h*)] plays an important role in determining *g*^(2)^. In Fig. 6 ▸(*b*), *t*_transit_ is considerably larger than the decay of *g*^(2)^, which means that the system is given enough time to decorrelate by diffusion before the sample moves out of the beam. However, when *t*_transit_ is small enough to interfere with *g*^(2)^, for instance in Figs. 6 ▸(*e*) and 6(*h*), the correlation functions experience forced decorrelation that is faster than that given by Brownian motion. In addition, *t*_transit_ limits the maximum delay times that we can access since the illuminated sample volume is completely different after *t*_transit_. Despite the deviations of *g*^(2)^ between Brownian and combined motions, the dispersion relations (Γ as a function of *q*) are remarkably consistent. The extracted *D*_0,B_ and *D*_0,C_ show excellent agreements for all scenarios when the offset α is taken into account for the combined motions. In accordance with expectations, we found higher α values for faster drift motions and, consequently, higher transit relaxations (Γ_transit_ = 1/*t*_transit_), indicated as black horizontal lines in Figs. 6 ▸(*c*), 6(*f*) and 6(*i*).

**Figure 7:**
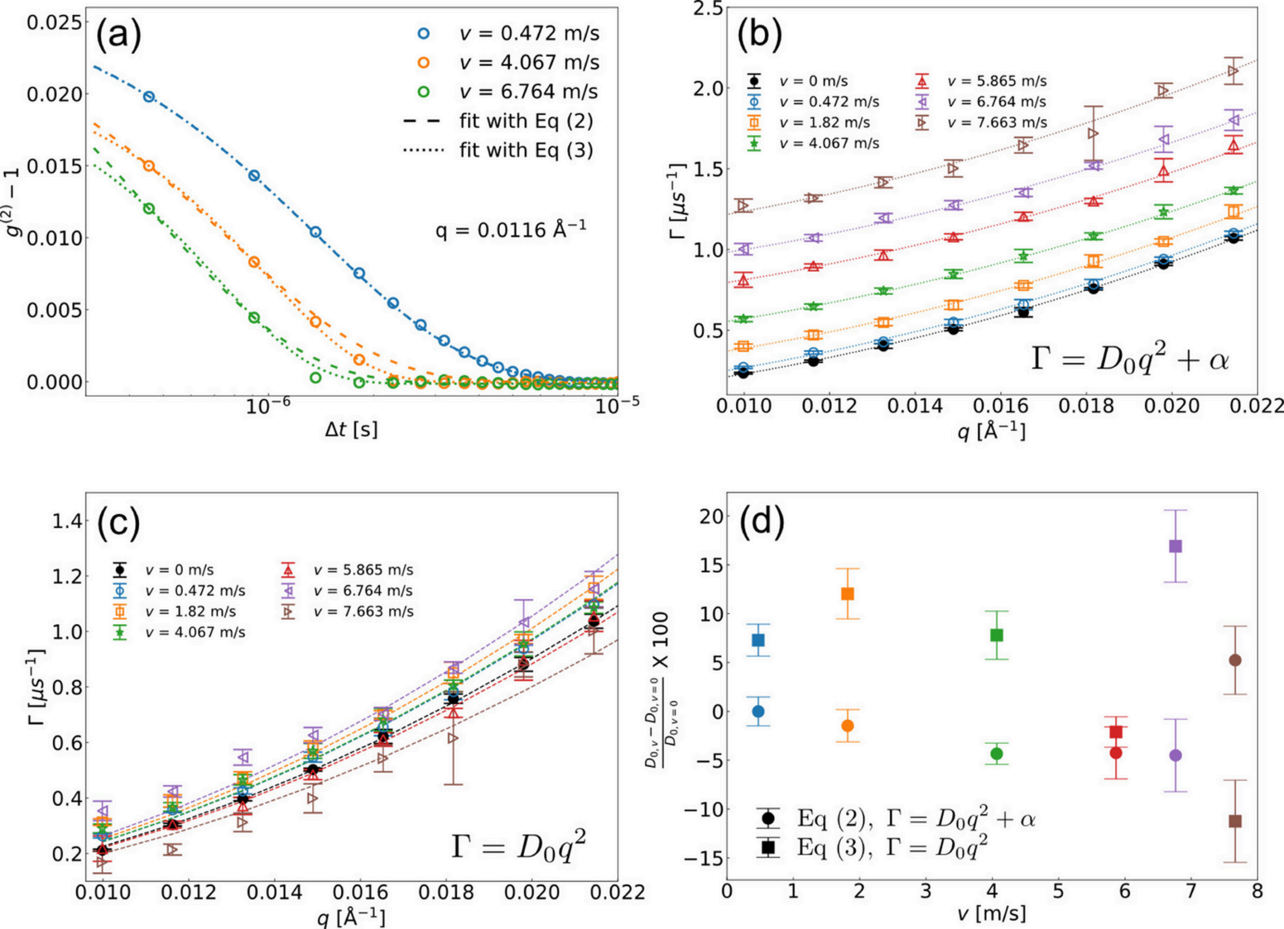
(*a*) Simulated *g*^(2)^ at the same *q* for varying *v*. The dashed and dotted lines represent the fitting curves of equations (2) and (3) and the corresponding result of Γ is shown in (*b*) and (*c*), respectively. (*d*) The errors in percent for the two data evaluation processes.

This simulation result can be also explained by the model taking into account continuous translation motions and the Gaussian shape of the beam given by (Chowdhury *et al.*, 1984 ▸) 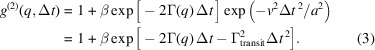
Note that the additional decaying term in equation (3) determined by the sample movement yields a faster-decaying behavior of *g*^(2)^ because of the reduction of sample spatial overlap over the delay. In addition, the behavior is independent of *q* and azimuthal scattering angles relative to the translation direction (Lurio *et al.*, 2021 ▸). We employed two different approaches in order to extract *D*_0_ from the simulation data. The first approach considers the sample movement in the step of fitting Γ using Γ = *D*_0_*q*^2^ + α, and Γ is the fitting result of equation (2). The second method considers equation (3) directly and evaluates *D*_0_ without taking α into account. Fig. 7 ▸(*a*) shows a comparison of the fitting results employing the two different approaches on simulated *g*^(2)^ data. The dashed and dotted lines denote the fitting lines employing equations (2) and (3), respectively, for varying translation speeds, *e.g.v* = 0.472, 4.067 and 6.764 m s^−1^. Equation (3) shows a better fitting result of *g*^(2)^ when the translation speed is faster but, in general, both models fit the correlation functions well. Fig. 7 ▸(*b*) shows the relaxation rates Γ obtained using equation (2) and the results of fitting with Γ = *D*_0_*q*^2^ + α. A faster *v* clearly provides a higher *y*-intercept α. In contrast, as equation (3) already takes into account *v*, the obtained relaxation rate Γ reflects the natural particle dynamics without any contamination from *v*, so the simple dispersion relation Γ = *D*_0_*q*^2^ is used to extract the diffusion coefficient *D*_0_ in Fig. 7 ▸(*c*). Fig. 7 ▸(*d*) displays the percent errors δ = [(*D*_0,*v*_ − *D*_0,*v*=0_)/*D*_0,*v*=0_] × 100 of the two different approaches as a function of *v*. Despite the different data evaluation processes, the extracted diffusion coefficients are in agreement within 10% error.

In order to reproduce the experimental result in Fig. 4 ▸, we took into account effective drift speed *v*_*r*_ considering two directional drift motions [*v*_*r*_ = 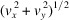]. The optimized simulation results of *v*_*r*_, *v*_*x*_ and *v*_*y*_ are 6.04, 4.49 and 4.04 m s^−1^, respectively. The obtained *D*_0,B_ and *D*_0,C_ are 22.6 and 22.1 µm^2^ s^−1^, respectively, together with α of 0.61 µs^−1^. The values are consistent with our experimental result, indicating the droplet moving about 4 m s^−1^ in both directions with the levitation device. Note that we have chosen the first approach, *i.e.* using equation (2) and Γ = *D*_0_*q*^2^ + α, to analyze the experimental and simulation results. We found that equation (3) was not adequate to describe the dynamics of *g*^(2)^ in Fig. 4 ▸(*b*), and the outcome of the fits was unreliable. We attribute this to the model system employed to derive equation (3) that assumed a constant movement of the system. In our experiment, the velocity of the levitated droplet is expected to include multiple time-dependent vector components, which are outside of the model assumptions given by Chowdhury *et al.* (1984 ▸). However, the simple approach of including *q*-independent offset to the relaxation rates Γ(*q*) [equation (2)] is describing the experimental data well.

## 5. Conclusion

We have demonstrated MHz XPCS with a containerless sample holder at the European XFEL. A millimetre-sized liquid droplet was levitated via acoustic pressure and decreased its solvent volume by evaporation with time. The volume fractions of silica nanoparticles dispersed in water were estimated by the structure factor of the SAXS patterns, changing from 0.83% to 9% during an evaporation time of 960 s. Considering the offset α of the dispersion relations allows us to capture the nanoscale diffusive dynamics and isolate them from the spatial jittering of the droplet in the levitator. In this experiment, the combined sample movement speed of both directions was estimated to be about 6 m s^−1^, but this is still compatible with accurate measurement of fast dynamics by MHz XPCS. This is a mode that currently only the European XFEL can offer. The dynamics obtained from a levitated droplet, from a sample filled into a quartz capillary, and in a simulation taking constant movement of the sample into account show excellent agreement. This successful demonstration represents a significant step toward expanding the use of X-ray speckle techniques, such as XPCS, XSVS and XCCA, for probing the dynamics of metastable states in soft-matter systems. By establishing the feasibility of MHz XPCS with a levitated droplet, our work opens new possibilities for studying non-equilibrium processes and phase transitions in a contactless environment, offering unique insights that are otherwise challenging to obtain with conventional sample environment.
